# Dapagliflozin combined with metformin improves blood glucose, bone metabolism and bone mineral density in elderly patients with type 2 diabetes mellitus complicated with osteoporosis

**DOI:** 10.1002/kjm2.12937

**Published:** 2025-01-15

**Authors:** Haiyan Zheng, Qian Wang, Min Si

**Affiliations:** ^1^ Department of Endocrinology Renmin Hospital, Hubei University of Medicine Shiyan Hubei China

**Keywords:** dapagliflozin, glycemic parameters, metformin, osteoporosis, type 2 diabetes mellitus

## Abstract

The incidence of type 2 diabetes mellitus (T2DM) complicated with osteoporosis (OP) (T2DM‐OP) is growing. Dapagliflozin and metformin are commonly prescribed to manage glycemic levels in T2DM patients. We investigated the clinical efficacy of combining dapagliflozin with metformin in elderly patients with T2DM‐OP. Totally 144 T2DM‐OP patients were prospectively enrolled and allocated into two groups: the Metformin and Dapagliflozin + Metformin groups. Each group received treatment for 12 months. Fasting peripheral blood samples were collected before and after 12 months of treatment. Glycemic parameters and bone metabolic parameters were measured using oral glucose tolerance test, automatic biochemical analyzers, or liquid chromatography. Bone mineral density (BMD) changes at lumbar vertebrae (L1‐4), femoral neck (FN) and total hip (TH) were assessed using dual‐energy X‐ray bone mineral densitometry. Pain severity was evaluated using the visual analog scale (VAS). The total effective rate, fracture incidence, and adverse reaction rate were also evaluated. After 12 months, both groups showed improvements in glycemic parameters, bone metabolic parameters, and BMD at L1‐4, FN, and TH, and reductions in VAS scores. The Dapagliflozin + Metformin group exhibited more significant improvements. The overall effective rate was higher and fracture incidence, was lower in Dapagliflozin + Metformin group, with comparable rates of adverse reactions and safety profiles between the two groups. Taken together, treatment with a combination of dapagliflozin and metformin led to improvements in blood glucose levels, bone metabolism, and BMD in elderly patients with T2DM‐OP, demonstrating superior efficacy and safety compared to metformin monotherapy.

## 1 INTRODUCTION

Diabetes mellitus (DM) is a chronic metabolic disorder characterized by persistent hyperglycemia resulting from impaired insulin secretion, insulin resistance, or a combination of both. Type 2 diabetes mellitus (T2DM) accounts for 90%–95% of all DM cases globally.^1^, ^2^ Osteoporosis (OP), a progressive skeletal condition, is marked by decreased bone mineral density (BMD), bone mass, and calcium levels, as well as microarchitectural deterioration of bone tissue. These changes increase the risk of fragility fractures, hospitalization, disability, and mortality, contributing significantly to the healthcare burden.^3^, ^4^ Emerging evidence highlights a strong association between T2DM and OP, where prolonged hyperglycemia and associated complications contribute to reduced BMD and increased fragility fracture risk.^5^, ^6^ The co‐occurrence of T2DM and OP (T2DM‐OP) is increasingly prevalent, particularly in aging populations, underscoring the need for effective therapeutic interventions to improve patient outcomes and reduce morbidity and mortality.^7^, ^8^

The shared pathophysiology of DM and OP suggests that certain antidiabetic agents may have dual benefits: improving glycemic control while mitigating bone loss and fracture risk. Metformin, a first‐line oral hypoglycemic agent widely used in the management of T2DM, improves insulin sensitivity, suppresses hepatic glucose production, and enhances peripheral glucose uptake.^9^ Beyond its glycemic benefits, metformin has shown promise in addressing bone metabolism dysfunctions in T2DM patients. Studies indicate that metformin attenuates bone mass loss by targeting mechanisms such as hyperglycemia‐induced tissue damage, accumulation of advanced glycosylation end products in collagen, insulin deficiency, reduced osteocalcin and insulin‐like growth factor‐1 levels, and chronic inflammation.^10^ These findings position metformin as a potential therapeutic option for T2DM‐OP; however, its efficacy in this context requires further clinical validation.

Dapagliflozin, a selective sodium–glucose cotransporter protein 2 inhibitor, lowers blood glucose levels by reducing renal glucose reabsorption and is widely used for the management of T2DM.^11^ Beyond glycemic control, dapagliflozin has demonstrated therapeutic benefits for various T2DM complications, including diabetic retinopathy, nephropathy, and cardiovascular disorders.^12^ Despite its established efficacy in managing hyperglycemia and its complications, the effects of dapagliflozin on bone metabolism in patients with T2DM‐OP remain unclear.

Preclinical studies suggest that dapagliflozin may have a protective role in bone health under certain conditions. For instance, in Zucker Diabetic Fatty rats, dapagliflozin alleviated hyperglycemia‐associated osteoporosis by reducing hypercalciuria, a condition linked to excessive urinary calcium excretion.^13^ Additionally, clinical research by Ljunggren et al. indicated that dapagliflozin monotherapy did not adversely impact markers of bone resorption, bone formation, or BMD in T2DM patients with inadequate glycemic control on metformin.^14^ However, these findings do not fully elucidate the potential synergistic effects of dapagliflozin when combined with metformin in elderly T2DM‐OP patients.

Given the increasing prevalence of T2DM‐OP and its associated healthcare burden, understanding the dual benefits of dapagliflozin and metformin in this population is critical. This study aims to evaluate the combined effects of dapagliflozin and metformin on glycemic parameters, bone metabolism, and BMD in elderly T2DM‐OP patients, providing insights into their clinical utility and guiding future therapeutic strategies.

## 2 MATERIALS AND METHODS

### 2.1 Ethics statement

This study was approved by the Ethics Committee of our hospital and conducted in compliance with the Declaration of Helsinki. Prior to participation, all patients voluntarily provided written informed consent after a comprehensive explanation of the study's objectives, procedures, potential risks, and benefits.

### 2.2 Sample size estimation and effect size and statistical efficacy calculation

Sample size estimation was performed using G*Power 3.0.10 (University of Düsseldorf, Nordrhein‐Westfalen, Germany). The test method used was a *t*‐test (two‐tailed) with parameters set at *α* = 0.05, 1 − *β* = 0.8, effect size (*d*) = 0.5, and an allocation ratio of N1 (Metformin group):N2 (Dapagliflozin + Metformin group) = 1:1.^15^ The analysis indicated that a minimum sample size of 128 cases was required. Accounting for a potential 10% attrition rate, an initial sample size of 144 cases was recruited. There were no instances of withdrawal or loss to follow‐up, resulting in a final cohort of 144 participants.

Post‐treatment, effect sizes for key bone metabolic indicators, including 25‐hydroxy‐vitamin D3 [25‐(OH)D3], human bone alkaline phosphatase (BALP), and osteoprotegerin (OPG), were calculated using the formula: effect size (*d*) = difference in mean values between groups/pooled standard deviation. The calculated effect sizes 25‐(OH)D3, BALP, and OPG were 1.30, 1.11, and 1.55, respectively, all exceeding the predetermined threshold of 0.5. Statistical power (1 − *β*) was subsequently assessed using G*Power 3.0.10, yielding values of 1.00 for all three indicators, surpassing the expected minimum statistical efficacy of 0.80. The results confirm that the study sample size was adequate and provided robust statistical efficacy.

### 2.3 Study subjects and clinical data

This prospective study enrolled 144 patients diagnosed with T2DM‐OP from January 2021 to June 2022. Participants were randomly assigned to either the Metformin group or the Dapagliflozin + Metformin group using a random number table, with 72 patients in each group.

**FIGURE 1:**
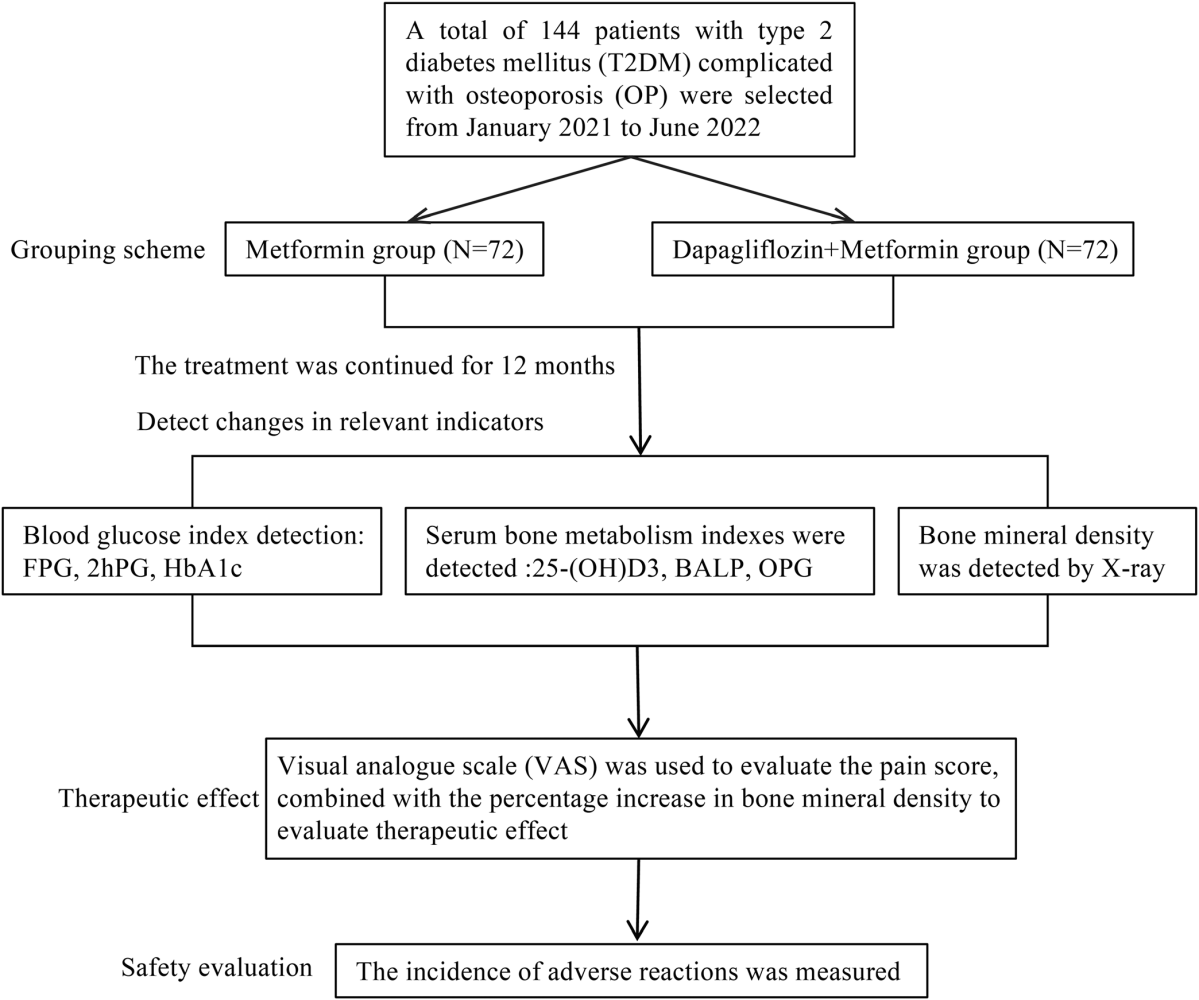
Study flowchart.

Baseline characteristics, including age, sex, body mass index (BMI), history of fractures, supplementation of calcium and vitamin D, were recorded. The Metformin group comprising of 31 males and 41 females, aged 60–75 years (mean 67.8 ± 4.9 years), with BMI ranging from 17.8 to 28.6 (mean 23.9 ± 3.2). In comparison, the Dapagliflozin + Metformin group included 34 males and 38 females, aged 60–75 years (mean 67.9 ± 4.4 years), with BMI ranging from 18.2 to 29.6 (mean 24.3 ± 3.4) (Table 1). The study design is illustrated in Figure 1.

**TABLE 1:** Analyses of baseline data in the Metformin and Dapagliflozin + Metformin groups.

Indicators	Metformin group (*n* = 72)	Dapagliflozin + Metformin group (*n* = 72)	*p* value
Age (years)	67.8 ± 4.9	67.9 ± 4.4	0.8977
Sex (male/female)	31/41	36/38	0.6154
BMI	23.9 ± 3.2	24.3 ± 3.4	0.4685
Fracture history [*n* (%)]	23 (31.9%)	25 (34.7%)	0.7237
Pre‐treatment administration of calcium or vitamin D	26 (36.1%)	24 (33.3%)	0.7263
Insulin remedy	13 (18.1%)	11 (15.6%)	0.6547

*Note*: Measurement data were expressed as mean ± standard deviation, and independent sample *t* test was adopted for comparisons between the two groups. The counting data were expressed as number of cases (*n*), and Chi‐square test was utilized for comparisons between the two groups. *p* < 0.05 was accepted as indicative of significant differences.

Abbreviation: BMI, body mass index.

### 2.4 Inclusion and exclusion criteria

Participants were enrolled based on the following criteria: (1) individuals between 60 and 75 years of age; (2) confirmed first diagnosis of T2DM‐OP, with T2DM diagnosed based on criteria including random blood glucose ≥11.1 mmol/L, fasting plasma glucose (FPG) ≥ 7.0 mmol/L, and 2‐h postprandial glucose (2hPG) ≥ 11.1 mmol/L, and OP diagnosed in accordance with the Guidelines for the Diagnosis and Treatment of Primary Osteoporosis (2017), meeting one of the following criteria: (1) fragility fracture of the hip or vertebral body; (2) *T*‐value of midshaft BMD or BMD of the distal 1/3 of radius measured by dual‐energy X‐ray absorptiometry ≤−2.5; 3) BMD measurements consistent with low bone mass (−2.5 < *T*‐value < −1.0) + fragility fracture of the proximal humerus, pelvis, or distal forearm; (3) the first time to accept treatment for DM and OP; (4) provision of written informed consent.

Exclusion criteria included: (1) allergy to study drug; (2) presence of type I DM and secondary OP; (3) presence of hematologic lesions, malignant tumors, or other metabolic and endocrine disorders; (4) occurrence of cerebral, cardiac, pulmonary, hepatic, or renal abnormalities; (5) presence of psychiatric disorders; (6) recent use of medications affecting bone metabolism, such as glucocorticosteroids; and (7) poor compliance (medication taken <90%).

### 2.5 Treatment protocol

1. In the Metformin group, patients were prescribed metformin hydrochloride extended‐release tablets (0.5 g × 60 tablets/box, National Medicine Permission No.: H20178002, SinocorpPharma, Huizhou, Guangdong, China) to be taken orally twice daily, with each dose amounting to 500 mg. Additionally, patients received a daily oral administration of 5 mg placebo which was identical in appearance and method of administration to dapagliflozin tablets, but devoid of any pharmacological activity. Supplemental insulin therapy was adopted in cases where glycemic control with oral hypoglycemic agents proved ineffective. At the same time, patients were administered 70 mg alendronate sodium (Merck Sharp & Dohme S.p.A., Hangzhou, Zhejiang, China; National Medicine Permission No.: H20084179) once weekly, and 600 mg Calcium D (Haleon, Suzhou, Jiangsu, China; National Medicine Permission No.: H10950029, 30 tablets/bottle) once daily for the treatment of OP. The treatment protocol was carried out for 12 months continuously in a double‐blind manner.
2. In the Dapagliflozin + Metformin group, patients were administered dapagliflozin tablets orally (10 mg/1000 mg × 28 tablets/box, Import Registration Certificate No.: HJ20230080, National Medicine Permission No.: HJ20230080, Astra Zeneca Pharmaceuticals LP, Mount Vernon, IN, USA) once daily, with each dose amounting to 5 mg, alongside metformin hydrochloride as prescribed in the Metformin group. Supplemental insulin therapy was adopted in cases where glycemic control with oral hypoglycemic agents proved ineffective. Concurrently, the treatment for OP was conducted. The treatment protocol was carried out for 12 months continuously in a double‐blind manner.

### 2.6 Medication administration regimen

To enhance patient adherence and ensure therapeutic efficacy, a comprehensive medication regimen was designed in collaboration with the participants. The following strategies were implemented: (1) family members were encouraged to assist patients in adhering to the prescribed regimen, employing tools such as reminders and medication dispensers to reduce missed doses; (2) patients were provided with medication administration cards to record dosage intake and any observed side effects; (3) monthly follow‐ups or telephone check‐ins were conducted to monitor adherence, address concerns, and provide encouragement to improve compliance.

### 2.7 Blood sample collection

Peripheral blood samples (5 mL) were collected from all participants before treatment and after 12 months of treatment following an overnight fast. An oral glucose tolerance test was administered with blood samples collected 2 h after oral glucose administration. The blood samples were centrifuged at 300 × g for 10 min, and the resultant serum were carefully separated and stored at −20°C. Fasting serum samples were used to analyze levels of FPG, glycosylated hemoglobin (HbA1c), 25‐(OH)D3, BALP, and OPG. Postprandial serum (2 h after oral glucose intake) was used to assess 2hPG.

### 2.8 Indicator observation

All indicators were assessed in a double‐blind manner using the following methods:

1. Glycemic parameters: FPG and 2hPG were measured the hexokinase method on a Dr. Doctor DM5279 blood glucose tester (Dr. Doctor, Hangzhou, Zhejiang, China) to determine FPG and 2hPG levels. HbA1c concentration was determined using high‐performance liquid chromatography (HPLC). Standard curves were prepared using an HPLC system (600E‐2487, Waters Corporation, Milford, MA, USA) and a standard sample (NIM‐RM3626, National Institute of Metrology, Beijing, China). Blood samples were pretreated for hemoglobin extraction and purification before HbA1c quantification.
2. Bone metabolic indicators: Levels of 25‐(OH)D3, BALP, and OPG were analyzed using a chemiluminescence assay on a fully automatic biochemical analyzer (Roche 601, Roche Basel, Switzerland).
3. BMD assessment: BMD measurements were performed for lumbar vertebrae 1–4 (L1‐4), femoral neck (FN), and total hip (TH) using dual‐energy X‐ray absorptiometry (BMD‐A7, PinYuan Electronic Technology, Xuzhou, Jiangsu, China). Instrument calibration was conducted according to operation guidelines, with calibration repeated three times prior to testing.
4. Efficacy assessment: Pain was assessed using the visual analog scale (VAS), with scores ranging from 0 (no pain) to 10 (worst pain imaginable).^16^ Markedly effective: VAS score reduced by >3 points and BMD increased by ≥20%. Effective: VAS score reduced by 2 points, and BMD increased by 10%–20%. Ineffective: Cases that did not meet the above criteria. Total effective rate = (markedly effective cases + effective cases)/total cases × 100%.
5. Adverse reactions: The incidence of adverse reactions, such as nausea/vomiting, diarrhea/abdominal pain, hypoglycemia, rash and others, was recorded throughout the treatment period.

### 2.9 Statistical analysis

Statistical analysis was performed using SPSS 22.0 (IBM, Armonk, NY, USA). Normal distribution was assessed using the Shapiro–Wilk test. Measurement data conforming to normal distribution were expressed as mean ± standard deviation. One‐way analysis of variance was utilized for used for multiple‐group comparisons. Counting data were expressed as n (%), with differences analyzed using the Chi‐square test. Statistical significance was defined as *p* < 0.05.

## 3 RESULTS

### 3.1 Baseline data analyses

As shown in Table 1, there were no statistically significant differences in baseline characteristics, including age, sex ratio, BMI, fracture history, pre‐treatment administration of calcium or vitamin D, and use of insulin therapy between the Metformin and Dapagliflozin + Metformin groups (all *p* > 0.05). These findings indicate that the two groups were well‐matched and comparable at baseline.

### 3.2 Comparative analyses of glycemic parameters before and after treatment

Changes in glycemic parameters before and 12 months after treatment are presented in Table 2. Before treatment, no significant differences were observed between the two groups in FPG, 2hPG, and HbA1c levels (all *p* > 0.05). Following treatment, both groups demonstrated significant reductions in FPG, 2hPG, and HbA1c levels compared to their baselines values (all *p* < 0.05). The reductions in FPG, 2hPG, and HbA1c levels were significantly greater in the Dapagliflozin + Metformin group than in the Metformin group (all *p* < 0.05).

**TABLE 2:** Comparative analyses of blood glucose indicators before and after treatment in the Metformin and Dapagliflozin + Metformin groups.

Blood glucose indicator	Group	Before treatment	After 12 months of treatment	Delta value (∆)
FPG (mmol/L)	Metformin group (*n* = 72)	9.44 ± 1.44	7.15 ± 0.91^a^	−2.29 ± 0.87
	Dapagliflozin + Metformin group (*n* = 72)	9.36 ± 1.49	6.40 ± 0.92^ab^	−2.97 ± 0.93
2hPG (mmol/L)	Metformin group (*n* = 72)	12.75 ± 1.24	9.11 ± 0.94^a^	−3.64 ± 1.24
	Dapagliflozin + Metformin group (*n* = 72)	12.84 ± 1.35	8.51 ± 0.80^ab^	−4.33 ± 1.21
HbA1c (mmol/L)	Metformin group (*n* = 72)	8.83 ± 1.12	6.77 ± 0.86^a^	−2.06 ± 0.76
	Dapagliflozin + Metformin group (*n* = 72)	8.87 ± 1.10	6.22 ± 0.78^ab^	−2.65 ± 0.68

*Note*: Measurement data were expressed as mean ± standard deviation. Independent sample *t* test was applied for comparisons between the two groups. Difference was regarded as statistically significant at *p* < 0.05. ‘a’ indicated *p* < 0.05 when compared to pre‐treatment, and ‘b’ indicated *p* < 0.05 when compared to the Metformin group.

Abbreviations: 2hPG, 2 h plasma glucose; FPG, fasting plasma glucose; HbA1c, glycosylated hemoglobin.

### 3.3 Comparative analyses of serum bone metabolic indicators before and after treatment

Table 3 summarizes the serum bone metabolic indicator levels [25‐(OH)D3, BALP, and OPG] before and after treatment. Before treatment, no significant differences were noted in 25‐(OH)D3, BALP, or OPG levels between the two groups (all *p* > 0.05). After treatment, both groups showed significant improvements in serum bone metabolic indicators, with an elevation in 25‐(OH)D3 and OPG levels and a reduction in BALP levels compared to baseline (all *p* < 0.05). The Dapagliflozin + Metformin group exhibited more pronounced improvements in 25‐(OH)D3 and OPG levels compared to the Metformin group (all *p* < 0.05).

**TABLE 3:** Comparative analyses of serum bone metabolism indicators before and after treatment in the Metformin and Dapagliflozin + Metformin groups.

Bone metabolism indicators	Group	Before treatment	After12 months of treatment	Delta value (∆)
25‐(OH)D3 (ng/mL)	Metformin group (*n* = 72)	14.58 ± 1.45	20.57 ± 1.70^a^	5.98 ± 1.38
	Dapagliflozin + Metformin group (*n* = 72)	14.27 ± 1.60.	23.63 ± 2.06^ab^	9.35 ± 2.43
BALP (IU/L)	Metformin group (*n* = 72)	34.83 ± 4.63.	31.17 ± 4.45^a^	−3.66 ± 1.42
	Dapagliflozin + Metformin group (*n* = 72)	34.20 ± 4.61	28.09 ± 4.03^ab^	−6.11 ± 2.21
OPG (pg/mL)	Metformin group (*n* = 72)	187.41 ± 36.04	238.45 ± 41.53^b^	51.04 ± 14.87
	Dapagliflozin + Metformin group (*n* = 72)	189.08 ± 36.61	280.87 ± 45.62^ab^	91.78 ± 18.11

*Note*: Measurement data were expressed as mean ± standard deviation. Independent sample *t* test was applied for comparisons between the two groups. Differences were considered to be statistically significant at *p* < 0.05. ‘a’ indicated *p* < 0.05 when compared to pre‐treatment, and ‘b’ indicated *p* < 0.05 when compared to the Metformin group.

Abbreviations: 25‐(OH)D3, 25‐hydroxy‐vitamin D3; BALP, human bone alkaline phosphatase; OPG: osteoprotegerin.

### 3.4 Comparative analyses of BMD before and after treatment

As shown in Table 4, baseline BMD measurements at the L1‐4, FN, and TH did not differ significantly between the Metformin group and the Dapagliflozin + Metformin group (all *p* > 0.05). After 12 months of treatment, both groups experienced a significant increase in BMD at all measured sites compared to their baseline values (all *p* < 0.05). The increase in BMD was significantly greater in the Dapagliflozin + Metformin group than in the Metformin group (all *p* < 0.05) (Table 4).

**TABLE 4:** Comparative analyses of BMD before and after treatment in the Metformin and Dapagliflozin + Metformin groups.

BMD	Group	Before treatment	After 12 months of treatment	Delta value (∆)
L1‐4 (g/cm^2^)	Metformin group (*n* = 72)	0.724 ± 0.079	0.774 ± 0.080^a^	0.050 ± 0.016
	Dapagliflozin + Metformin group (*n* = 72)	0.720 ± 0.082	0.809 ± 0.094^ab^	0.089 ± 0.019
FN (g/cm^2^)	Metformin group (*n* = 72)	0.629 ± 0.055	0.678 ± 0.065^a^	0.049 ± 0.014
	Dapagliflozin + Metformin group (*n* = 72)	0.623 ± 0.053	0.711 ± 0.052^ab^	0.088 ± 0.019
TH (g/cm^2^)	Metformin group (*n* = 72)	0.659 ± 0.064	0.712 ± 0.065^a^	0.053 ± 0.011
	Dapagliflozin + Metformin group (*n* = 72)	0.654 ± 0.064	0.754 ± 0.72^ab^	0.100 ± 0.015

*Note*: Measurement data were expressed as mean ± standard deviation. Comparisons between the two groups were performed by independent sample *t* test. *p* < 0.05 was considered statistically significant. ‘a’ indicated *p* < 0.05 when compared to pre‐treatment, and ‘b’ indicated *p* < 0.05 when compared to the Metformin group.

Abbreviations: BMD, bone mineral density; FN, femoral neck; L1‐4, Lumbar vertebrae 1–4; TH, total hip.

### 3.5 VAS score and overall efficacy analysis

Table 5 displays the changes in VAS scores and treatment efficacy. Before treatment, no significant differences were observed in VAS scores between the two groups (all *p* > 0.05). After treatment, VAS scores decreased significantly in both groups (all *p* < 0.05), with the Dapagliflozin + Metformin group showing a significantly greater reduction than the Metformin group (all *p* < 0.05). The overall effective treatment rate was markedly higher in the Dapagliflozin + Metformin group compared to the Metformin group (94.40% vs. 79.20%, *p* = 0.0068) (Table 6).

**TABLE 5:** VAS score analyses of the Metformin and Dapagliflozin + Metformin groups.

Group	VAS score before treatment	VAS score after 12 months of treatment	Delta value (∆)
Metformin group (*n* = 72)	7.37 ± 1.77	4.16 ± 0.85^a^	−3.21 ± 1.01
Dapagliflozin + Metformin group (*n* = 72)	7.34 ± 1.79	3.36 ± 0.82^ab^	−3.98 ± 1.10

*Note*: Measurement data were expressed as mean ± standard deviation, and comparisons between two groups were done by independent sample *t* test. Difference was considered statistically significant at *p* < 0.05. ‘a’ indicated *p* < 0.05 when compared to pre‐treatment, and ‘b’ indicated *p* < 0.05 when compared to the Metformin group.

Abbreviation: VAS, visual analog score.

**TABLE 6:** Analyses of the efficacy of the Metformin and Dapagliflozin + Metformin groups.

Group	Ineffective	Effective	Markedly effective	Overall effective rate
Metformin group (*n* = 72)	15 (20.8%)	23 (32%)	34 (47.2%)	57 (79.20%)
Dapagliflozin + Metformin group (*n* = 72)	4 (5.6%)	20 (27.7%)	48 (66.7%)	68 (94.40%)
*p* value				0.0068

*Note*: The counting data were expressed as the number of cases (*n*) and percentage (%). Chi‐square test was applied for comparisons between the two groups. *p* < 0.05 was considered statistically significant.

### 3.6 Analysis of fracture incidence during follow‐up

During the 12‐month follow‐up period, fracture incidence was analyzed (Table 7). The fracture rate in the Dapagliflozin + Metformin group was significantly lower compared to the Metformin group (4.2% vs. 13.8%, *p* = 0.0418).

**TABLE 7:** Analysis of fracture incidence in patients during follow‐up.

Group	Metformin group (*n* = 72)	Dapagliflozin + Metformin group (*n* = 72)	*p* value
Fracture incidence [*n* (%)]	10 (13.8%)	3 (4.2%)	0.0418

*Note*: The counting data were expressed as number of cases (*n*) and percentages (%), and Chi‐square test was utilized for comparisons between the two groups. *p* < 0.05 was indicative of significant differences.

### 3.7 Analysis of adverse reactions

Adverse reactions, including nausea/vomiting, diarrhea/abdominal pain, hypoglycemia, and rash, were recorded during the 12‐month treatment period. No severe adverse events were reported in either group. The overall incidence of adverse reactions was comparable between the two groups (16.7% in the Dapagliflozin + Metformin group vs. 13.9% in the Metformin group, *p* = 0.6432) (Table 8).

**TABLE 8:** Analyses of adverse reactions in the Metformin and Dapagliflozin + Metformin groups.

Group	Nausea/vomiting	Diarrhea/abdominal pain	Hypoglycemia	Rash	Adverse reactions
Metformin group (*n* = 72)	3 (4.2%)	2 (2.8%)	3 (2.8%)	2 (2.8%)	10 (13.92%)
Dapagliflozin + Metformin group (*n* = 72)	3 (4.2%)	3 (4.2%)	4 (5.6%)	2 (2.8%)	12 (16.7%)
*p* value					0.6432

*Note*: The counting data were expressed as the number of cases (*n*) and percentage (%). Chi‐square test was adopted for comparisons between the two groups. *p* < 0.05 was considered statistically significant.

## 4 DISCUSSION

T2DM disrupts multiple metabolic pathways, including those involved in bone remodeling, thereby increasing the risk of OP. Studies estimate that T2DM patients are one to three times more likely to develop OP compared to non‐diabetic individuals of the same age.^17^ Current management strategies for T2DM‐associated OP focus on addressing both the underlying diabetes and bone health, often incorporating hypoglycemic agents alongside anti‐osteoporotic treatments.^18^ In this context, our study evaluated the clinical efficacy of dapagliflozin combined with metformin in elderly T2DM‐OP patients.

Glycemic markers, including HbA1c, FPG, and 2hPG, are critical indicators of glycemic control. These markers significantly decline with effective treatment in T2DM patients,^19^ and are also used to identify those at risk of diabetes development.^20^ Metformin, as a first‐line oral hypoglycemic agent, is well‐recognized for its ability to reduce hepatic gluconeogenesis, leading to decreased HbA1c and FPG levels.^21^, ^22^ However, given the complex pathophysiology of T2DM, combination therapy therapies are often required to achieve optimal glycemic control.^23^ For example, metformin combined with repaglinide has demonstrated superior efficacy and safety over monotherapy.^24^

Dapagliflozin, a sodium‐glucose cotransporter‐2 inhibitor, offers additional benefits beyond glycemic control, including positive effects on renal function and cardiovascular risk in T2DM patients.^25^ However, the specific benefits of dapagliflozin combined with metformin for T2DM‐OP patients are less well‐defined. Our findings indicate that while both metformin monotherapy and combination therapy with dapagliflozin effectively reduce FPG, 2hPG, and HbA1c levels, combination therapy demonstrates superior glycemic improvements. This suggests that dapagliflozin may enhance metformin's glucose lowering effects in T2DM‐OP patients.

In addition to glycemic control, metformin has shown promise as an adjunct treatment for bone metabolism disorders.^10^ Dapagliflozin has also demonstrated metabolic and antihyperglycemic effects in type 1 DM.^26^ The role of bone metabolic markers further underscores the relevance of our study, as 25‐(OH)D3, the main circulating form of vitamin D, is frequently deficient in T2DM patients and correlates with OP onset and progression.^27^, ^28^ BALP, a marker of osteoblast activity, reflects bone formation but also indicates high bone turnover.^29^, ^30^ OPG, a soluble receptor that inhibits osteoclastogenesis, is often reduced in T2DM patients, leading to increased bone resorption and OP risk.^31^, ^32^ Combination therapy with metformin and alendronate has been shown to mitigate bone glucose metabolism disturbances and reduce bone loss.^33^ Similarly, our results demonstrate that both metformin monotherapy or combination therapy with dapagliflozin improve bone metabolic parameters by increasing 25‐(OH)D3 and OPG levels and decreasing BALP levels, with combination therapy showing superior outcomes. These findings suggest enhanced regulation of bone turnover and reduced OP risk in T2DM‐OP patients receiving dapagliflozin and metformin.

Patients with T2DM are at an elevated risk of vertebral and hip fractures, even when BMD measurements falls within the normal range.^34^ Interestingly, despite having higher BMD levels compared to healthy individuals, those with T2DM still experience an increased fracture risk, highlighting the complex interplay between diabetes and bone health.^35^ Animal studies, such as those by David Benaiges Boix et al. have shown that metformin improves BMD and counteracts the adverse effects of T2DM on adipogenesis.^36^ Additionally, prior research supports metformin's positive impact on bone metabolism and BMD in elderly T2DM patients, further demonstrating its therapeutic potential in this demographic.^37^

In our study, both metformin monotherapy and combination therapy with dapagliflozin led to significant increases in BMD at L1‐4, FN, and TH, with combination therapy showing superior improvements. This suggests that dapagliflozin enhances metformin's efficacy in improving BMD in T2DM‐OP patients. Furthermore, the combination treatment also significantly reduced fracture incidence compared to metformin monotherapy, aligning with findings from other studies that highlight the benefits of combination therapies for glycemic control and bone health.

Pain reduction, as assessed by VAS scores, and a higher overall effectiveness rate was also observed with combination therapy. These results are consistent with the efficacy and safety reported for fixed‐dose metformin and other SGLT‐2 inhibitors, such as canagliflozin, in T2DM management.^38^ Furthermore, improvements in glycemic parameters, including blood glucose and HbA1c levels, have been associated with a reduced prevalence of OP, underscoring the importance of optimizing glycemic control to enhance bone health.^39^

Our findings suggest that dapagliflozin combined with metformin provides a dual benefit in improving blood glucose control and bone health in elderly T2DM‐OP patients. This dual therapy offers a promising therapeutic approach, demonstrating efficacy and safety in this vulnerable population. By closely monitoring blood glucose indicators and bone health parameters, healthcare providers can improve early detection and management of T2DM‐OP, potentially reducing its incidence and improving treatment outcomes.

However, the study has limitations that must be acknowledged. The single‐center design and relatively small sample size limit the generalizability of our findings. Additionally, the study focused exclusively on individuals aged 60–75 years, which may introduce age‐related confounding factors and bias. Future studies should involve multi‐center trials with larger and more diverse populations to validate the findings further. Exploring the efficacy of dapagliflozin and metformin in different age groups and assessing long‐term outcomes would also provide a more comprehensive understanding of its clinical utility.

In conclusion, combining dapagliflozin with metformin appears to be a promising strategy for managing T2DM‐OP, offering superior glycemic control, improved bone metabolism, and enhanced BMD compared to monotherapy. These findings offer valuable insights for clinicians and emphasize the importance of integrated treatment approaches in managing this complex condition.

## CONFLICT OF INTEREST STATEMENT

The authors declare no conflict of interest.

## ETHICS STATEMENT

This study was approved by the Ethics Committee of Renmin Hospital, Hubei University of Medicine and conducted in compliance with the Declaration of Helsinki. Prior to participation, all patients voluntarily provided written informed consent after a comprehensive explanation of the study's objectives, procedures, potential risks, and benefits.

## Data Availability

The data that support the findings of this study are available from the corresponding author upon reasonable request.
